# 

**Published:** 1887-07

**Authors:** 


					﻿adaes syare kare aywte
Vol III
JULY ,1887.
No. 1
DANIEL'S
MEDICAL
JOURNAL
FE DANIEL MD Ediagge adaew 
Narthern Offye 1217 Mabre
Jauie Haew laknie maje 
				

